# Ketones and brain health: from metabolic rescue to metabolic flexibility

**DOI:** 10.3389/fnut.2026.1977189

**Published:** 2026-09-21

**Authors:** Antonio Paoli

**Affiliations:** Department of Biomedical Sciences, University of Padova, Padova, Italy

**Keywords:** beta-hydroxybutyrate, brain health, cognition, fasting, intermittent metabolic switching, ketone bodies, metabolic flexibility, monocarboxylate transporters

## Abstract

The human brain relies predominantly on glucose under habitual dietary conditions, but this dependence is not absolute. During fasting, prolonged exercise, carbohydrate restriction or ketogenic interventions, beta-hydroxybutyrate (BHB) and acetoacetate (AcAc) become available as cerebral fuels and metabolic signals. Most literature on ketones and cognition has emphasized mild cognitive impairment (MCI), Alzheimer’s disease (AD) and other neurodegenerative disorders, where impaired cerebral glucose utilization provides a rationale for ketone-based brain-energy rescue. This Mini Review retains this evidence as proof of concept but shifts the focus toward a broader preventive hypothesis: repeated short periods of mild physiological ketosis may represent a normal component of cerebral metabolic flexibility and contribute to long-term brain health before cognitive impairment develops. This view is consistent with intermittent metabolic switching proposed recently, whereby transitions between glucose-dominant and fat/ketone-supported metabolism activate adaptive pathways involving mitochondrial function, stress resistance, autophagy, antioxidant defense and neurotrophic signaling. Mechanistically, ketones are transported into the brain through monocarboxylate transporters, oxidized by neurons and glia, and may influence histone deacetylases, redox state, neuroinflammation, BDNF-related signaling and cellular resilience. The key question may therefore be not whether sustained ketosis is superior to glucose metabolism, but whether modern lifestyles have reduced the physiological occasions on which ketones normally rise. Future studies should quantify the frequency, amplitude and metabolic context of ketone excursions induced by fasting, time-restricted eating, exercise, carbohydrate restriction, medium-chain triglycerides (MCT) intake or exogenous ketones, and relate these patterns to brain energetics and long-term cognitive trajectories.

## 1 Introduction: from brain energy rescue to brain health

The adult human brain represents only a small fraction of body mass but accounts for a disproportionately large share of resting energy expenditure. Under conventional mixed-diet conditions, glucose is its predominant substrate. This observation has often been interpreted as if the brain were almost exclusively glucose-dependent. Physiologically, however, cerebral metabolism is more flexible. When hepatic fatty-acid oxidation increases and insulin declines, the liver generates acetoacetate (AcAc) and beta-hydroxybutyrate (BHB), which circulate to extrahepatic tissues and cross the blood–brain barrier through monocarboxylate transporters before being converted to acetyl-CoA and oxidized in the tricarboxylic-acid cycle (1–3).

This adaptation is central to human survival during prolonged food deprivation because cerebral ketone utilization reduces the requirement for glucose and limits the need to generate glucose from gluconeogenic amino acids (4, 5). Nutritional ketosis should therefore be distinguished from diabetic ketoacidosis: the former is a regulated physiological state characterized by moderate ketone availability, whereas the latter is a pathological condition associated with severe insulin deficiency, uncontrolled lipolysis and metabolic acidosis (6).

The renewed interest in ketones and brain health has been driven mainly by evidence that cerebral glucose utilization is impaired in aging, MCI and AD (7–9), while the brain’s ability to take up and oxidize ketones is relatively preserved (10–12). This has led to the brain-energy-rescue model (13), in which ketones partially bypass defective glucose metabolism. Yet a review devoted only to MCI and AD risks missing a broader physiological question. If ketones are normal metabolites produced during fasting, exercise and carbohydrate restriction, their relevance may not be limited to treating an already impaired brain. They may also be part of a preventive metabolic repertoire through which the brain periodically experiences, and adapts to, changes in whole-body fuel availability.

## 2 Ketone metabolism, transport and cerebral metabolic flexibility

Ketogenesis occurs predominantly in hepatic mitochondria when fatty-acid availability and beta-oxidation increase while circulating insulin is relatively low. Acetyl-CoA generated from fatty acids is converted to AcAc, which can then be reduced to BHB. The liver exports these ketone bodies because it lacks succinyl-CoA: 3-ketoacid CoA transferase (SCOT), the enzyme required for terminal ketone oxidation. Extrahepatic tissues, including the brain, express the ketolytic machinery needed to reconvert ketones to acetyl-CoA (5).

The cerebral contribution of ketones increases broadly in proportion to their plasma availability. This does not imply an all-or-none switch from glucose to ketones. Glucose and ketones are oxidized simultaneously, with the relative contribution of ketones increasing during fasting, ketogenic diets, MCT supplementation or exogenous ketone administration (4, 11, 14, 15). Thus, the relevant concept is not fuel replacement, but metabolic flexibility. A crucial determinant of this flexibility is substrate transport. Because ketone bodies are hydrophilic monocarboxylates, their passage across the blood–brain barrier and cell membranes requires proton-linked monocarboxylate transporters (MCTs), which also transport lactate and pyruvate (16). In this mini-review, MCT refers to medium-chain triglycerides, whereas monocarboxylate transporters are referred to collectively as MCTs or identified by their specific isoforms (e.g., MCT1, MCT2, and MCT4). MCT1 is strategically located in endothelial cells of cerebral microvessels and facilitates the transfer of circulating monocarboxylates from blood to brain. MCT2, a high-affinity transporter with predominant neuronal localization in rodent brain, supports neuronal uptake, whereas MCT4 is mainly associated with astrocytes and monocarboxylate efflux. Experimental evidence indicates that ketogenic diet increases brain MCT1 expression, suggesting that sustained ketone availability is accompanied by an adaptive increase in the cerebral capacity to import and utilize monocarboxylates (17). Thus, adaptation to ketosis should not be viewed merely as an increase in circulating ketone concentrations, but as a coordinated remodeling of substrate transport and utilization. This transporter-level plasticity (18) provides a mechanistic basis for the progressive increase in cerebral ketone oxidation observed during fasting or ketogenic interventions and reinforces the concept that brain fuel selection is dynamic, regulated and highly dependent on substrate availability (19). In this context, MCTs expression may represent an important molecular component of cerebral metabolic resilience, allowing the brain to increase its reliance on ketone bodies when glucose availability or glucose utilization becomes limited.

This concept is central to the idea of cerebral metabolic flexibility. Ketogenic interventions do not need to eliminate glucose metabolism to be useful. Rather, ketones can supplement cerebral energy production when glucose uptake or utilization becomes inadequate. This is particularly relevant in aging and neurodegeneration (20), where the problem may be less an absolute shortage of systemic glucose than an impaired capacity of vulnerable brain regions to use it efficiently.

## 3 BHB as a fuel and signaling metabolite

The interpretation of ketosis exclusively as an energetic phenomenon is incomplete. BHB is an oxidizable substrate, but it is also a signaling metabolite capable of linking nutrient availability to cellular adaptation. Among the best-characterized mechanisms is modulation of histone deacetylase activity and downstream changes in histone acetylation and gene transcription (21–23). Although the physiological importance of individual epigenetic effects depends on concentration, cell type and experimental context, the broader principle is compelling: a metabolite generated during fasting, exercise or carbohydrate restriction can transmit information about whole-body energy status to cellular regulatory systems.

BHB also intersects with pathways involved in oxidative stress and mitochondrial resilience. Ketone metabolism can influence NAD+/NADH balance, reactive oxygen species production and antioxidant defense programs (5, 24, 25). These actions are relevant in the brain, where high oxygen consumption and abundant oxidizable lipids create vulnerability to oxidative damage. BHB may also influence inflammatory tone. Experimental work has shown that BHB can suppress NLRP3 inflammasome activation independently of its role as a conventional energy substrate (26).

A further mechanistic link concerns brain-derived neurotrophic factor (BDNF), which supports neuronal survival, synaptic plasticity, learning and memory. Sleiman and colleagues demonstrated that BHB can promote BDNF expression through mechanisms involving class I histone deacetylases (27), while Marosi and colleagues showed also the involvement of NFkb and histone acetyltransferase p300/EP300 in BDNF gene expression (28). More recently, a short ketogenic intervention was reported to increase global cerebral blood flow and circulating BDNF in humans (29). These findings do not prove that every dietary strategy producing ketones will improve cognition, but they strengthen the idea that BHB participates in signaling networks relevant to brain maintenance.

The most useful conceptual model is therefore not to view ketones as fuel versus ketones as signals, but rather as part of an integrated framework in which these two roles are closely intertwined. Oxidation of ketones modifies energetic state, substrate flux and redox balance, while BHB itself and metabolites downstream of its oxidation influence transcriptional, inflammatory and neurotrophic pathways. This dual role provides a plausible bridge between transient metabolic states and longer-term cellular adaptation.

## 4 Intermittent metabolic switching: a preventive framework

The preventive hypothesis proposed here draws heavily on the concept of intermittent metabolic switching developed by Mattson and colleagues. In this framework, repeated transitions between feeding and fasting shift metabolism from glucose use and energy storage toward fatty-acid mobilization, ketogenesis and cellular stress-resistance programs; refeeding subsequently supports growth, protein synthesis and repair (30–33). Importantly, the biological advantage is not necessarily continuous ketosis. Rather, it may reside in the capacity to enter and exit ketosis efficiently.

We recently (5)) discussed how fasting and ketogenic diets share several molecular features but are not identical interventions. Both can increase ketone availability and modify nutrient-sensing pathways, yet fasting adds a broader nutrient-deprivation signal, whereas ketogenic diets may produce ketosis in the presence of adequate or even high energy intake. This distinction is central for brain health. If BHB is partly a signal of altered energy availability, then the context in which it rises matters. A modest BHB excursion after an overnight fast, prolonged exercise or time-restricted eating does not convey the same physiological message as pharmacological ketone elevation in a carbohydrate-fed state, even though the molecule is chemically the same.

This suggests that brain health may benefit not from chronic high ketone concentrations, but from repeated low-to-moderate ketone excursions embedded in appropriate physiological contexts. Such excursions may act as a form of metabolic training, challenging neurons and glia to maintain mitochondrial flexibility, preserve MCTs-mediated substrate transport, engage antioxidant and autophagic pathways, and regulate neuroinflammatory tone. In this interpretation, mild ketosis is not primarily a treatment but an adaptive signal that periodically reminds the brain that energy availability is variable.

## 5 Mild ketosis as a brain-wellness signal: the central hypothesis

The central hypothesis of this Mini Review is that brief, repeated periods of mildly elevated ketones may contribute to brain wellness and prevention by maintaining cerebral metabolic flexibility. This differs from the therapeutic ketogenic model used in epilepsy or neurodegenerative disease. In established cerebral glucose hypometabolism, relatively high and sustained ketone availability may be desirable because ketones are used quantitatively to close an energy gap. In a metabolically healthy brain, however, there may be little rationale for chronically replacing glucose with ketones. The potential benefit may instead derive from preserving the biological machinery required to switch between substrates: hepatic ketogenesis, blood–brain-barrier transport through MCTs, neuronal ketolysis, mitochondrial oxidative capacity, and the signaling pathways responding to changes in energetic availability.

This preventive model also reframes the meaning of cognitive performance. Demonstrating that ketones improve a memory task in a healthy young adult over a few hours is not necessarily the most informative endpoint. If intermittent mild ketosis influences mitochondrial quality control, neuroinflammatory tone, BDNF-related signaling, oxidative-stress resistance and substrate flexibility, the main benefit may emerge over years as preserved neuronal metabolic competence rather than acute cognitive enhancement. In this sense, the target is not a nootropic effect but a resilience phenotype.

The distinction between ketone concentration and ketone dynamics is therefore important. Much ketogenic research implicitly assumes that greater or more sustained ketosis should produce greater biological benefit. For brain wellness, this assumption is unproven and may be misleading. Physiological BHB exposure is dynamic: concentrations rise during overnight fasting, increase further with prolonged fasting or carbohydrate restriction, may rise during prolonged exercise, and fall after carbohydrate-containing meals and insulin secretion. These oscillations may carry information about whole-body energy status. The relevant signal may be the frequency, amplitude and context of BHB excursions rather than a single fasting ketone value.

Exercise illustrates this point particularly well. It increases energetic demand while increasing systemic availability of alternative substrates, especially lactate and, in appropriate conditions, ketones. Both depend on MCTs-mediated transport, reinforcing the concept that cerebral metabolic flexibility is not a binary glucose-versus-ketone phenomenon. The mechanistic link between BHB and BDNF reported by Sleiman et al. (27)) does not mean that ketones mediate all cognitive benefits of exercise. However, it suggests that fasting, exercise, lactate metabolism, BHB signaling and neurotrophic adaptation may belong to a broader family of energetic challenges that promote neuronal resilience (32).

## 6 Clinical ketone interventions as proof of concept, not the whole story: from energy-gap hypothesis to the brain-rescue-hypothesis

Clinical studies in MCI, AD, and Parkinson’s disease remain highly informative, but they should be viewed primarily as proof of concept rather than as the whole story of ketone biology in the brain. Reduced cerebral glucose metabolism is one of the most reproducible metabolic abnormalities in AD (34) and may be detectable before advanced clinical symptoms become apparent (35). Regional reductions in glucose uptake are also observed in MCI and in individuals at increased risk of AD (36), while impaired glucose transport, insulin signaling, glycolytic flux, and mitochondrial function may all contribute to this metabolic vulnerability (37). These observations underpin the “brain energy-gap” hypothesis (13): if neuronal energy demand remains relatively stable while the ability to acquire or metabolize glucose declines, neuronal networks may become energetically constrained before irreversible neuronal loss is complete.

A central feature of this model is that the impairment appears to be at least partly substrate-specific. While cerebral glucose uptake declines in MCI and AD, the capacity to take up and oxidize ketone bodies appears comparatively preserved (10, 12). Accordingly, increasing circulating ketone availability may bypass rather than directly repair defective glucose utilization (14). This distinction is important because it avoids a simplistic interpretation of ketogenic therapy as a means of “replacing glucose.” The more physiologically plausible model is that ketones provide an additional oxidative substrate that partially closes an energy gap. The improvement can be obtained through exogenous ketones or via a physiological plasma increase of circulating ketone through ketogenic diet (38) or MCT’s supplementation (14, 20). These data are important because they link a measurable change in cerebral substrate utilization with changes in cognition rather than relying solely on peripheral ketone concentrations. More in detail in MCI, very-low-carbohydrate diets and ketogenic MCT drinks improved selected cognitive outcomes and increased cerebral ketone utilization while glucose uptake remained essentially unchanged (14, 20, 39, 40). In AD, MCT-based formulations and ketogenic diets have produced heterogeneous but encouraging results, with possible differences according to APOE genotype and disease stage (41–45). Evidence in Parkinson’s disease remains preliminary (40, 46, 47). These data support the brain-energy-rescue hypothesis [Cunnane et al., (13)], but they also imply something broader: the human brain can use ketones meaningfully when energetic conditions require it. For prevention, the question becomes whether regular, physiological opportunities to use this pathway help maintain the pathway itself.

This interpretation helps avoid a false dichotomy. A strict ketogenic diet, intermittent fasting, time-restricted eating, prolonged exercise, MCT supplementation and exogenous ketones can all raise BHB, but they do not reproduce the same metabolic state. A ketogenic diet reduces carbohydrate exposure and usually lowers insulin while increasing fatty-acid oxidation; fasting activates nutrient-deprivation pathways; exercise combines energetic demand with neuroendocrine and mechanical signals; MCTs stimulate hepatic ketogenesis by providing fatty-acid substrates even in the fed state; ketone salts and ketone monoesters can directly increase circulating BHB without requiring hepatic fatty-acid oxidation for ketone production, whereas some ketone diesters also provide medium-chain fatty acids that undergo hepatic oxidation and contribute to the resulting increase in BHB. Therefore, simply measuring whether BHB rises is insufficient. The same molecule may have different biological meaning depending on whether it appears with low insulin, energy deficit, exercise-induced lactate production, caloric restriction or exogenous ingestion (5, 30)) (see Figure 1).

**Figure 1:**
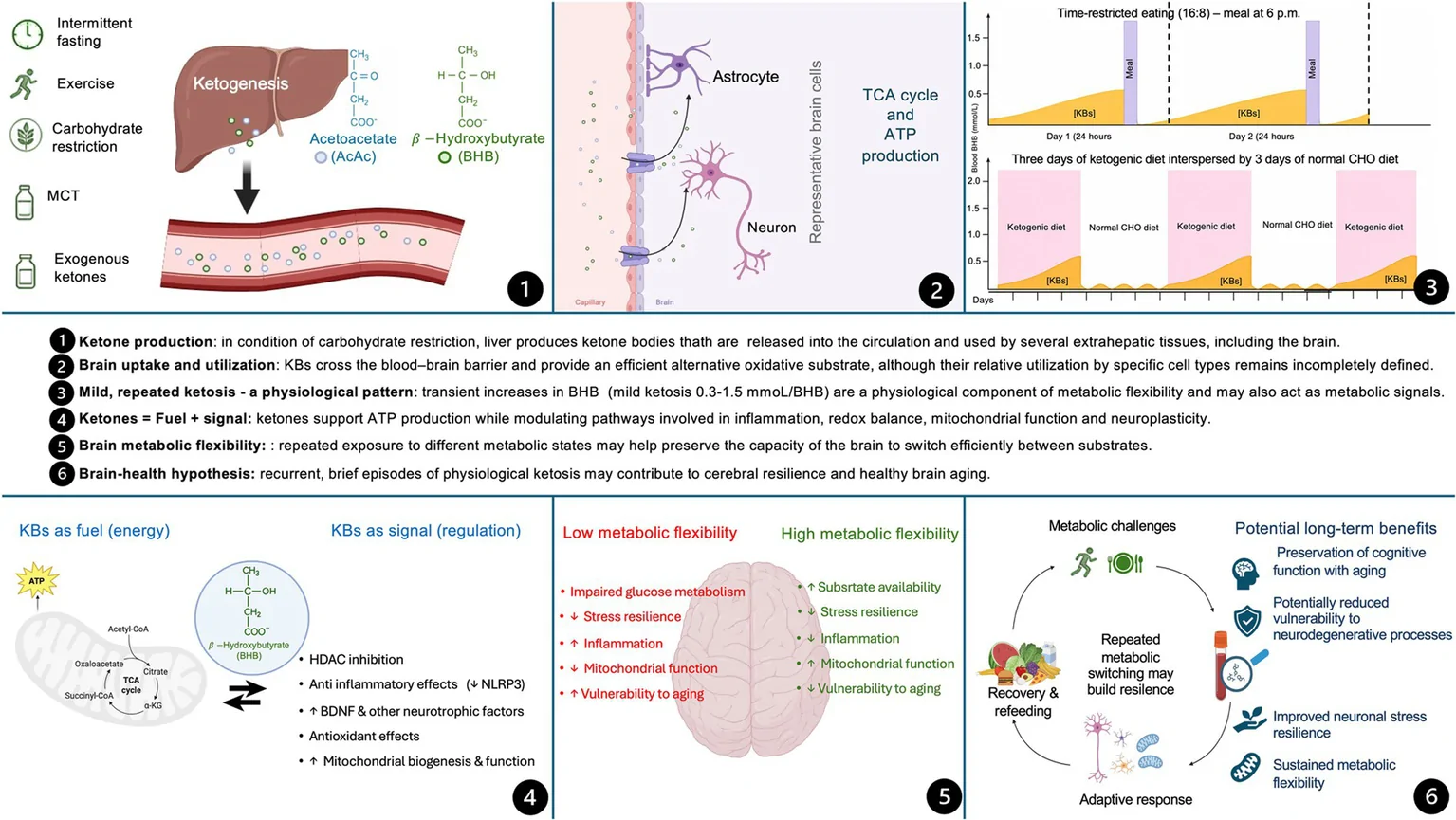
Proposed model linking physiological ketosis, cerebral metabolic flexibility, and brain health. Ketone bodies, mainly *β*-hydroxybutyrate (BHB) and acetoacetate (AcAc), are produced by the liver when carbohydrate availability and insulin signaling are reduced, as occurs during fasting, carbohydrate restriction, prolonged exercise, or following MCT intake. Once released into the circulation, ketone bodies cross the blood–brain barrier through monocarboxylate transport systems and can be oxidized by brain cells to support mitochondrial ATP production. Beyond their role as energy substrates, ketones, and particularly BHB, also act as signaling metabolites, influencing pathways involved in HDAC activity, NLRP3-mediated inflammation, oxidative stress, BDNF and neurotrophic signaling, and mitochondrial function. The figure proposes that brief and recurrent episodes of mild physiological ketosis may represent a form of metabolic switching that helps maintain cerebral metabolic flexibility. Rather than suggesting that chronic ketosis is required, the model hypothesizes that repeated transitions between glucose-dominant and ketone-supported metabolism may promote adaptive responses and neuronal resilience. Clinical studies showing preserved cerebral ketone utilization in conditions of impaired glucose metabolism provide proof of concept for this metabolic flexibility, whereas the possible long-term role of recurrent mild ketosis in maintaining brain health and reducing vulnerability to age-related metabolic dysfunction remains a hypothesis requiring prospective investigation. MCT, Medium-chain triglycerides; BHB, *β*-hydroxybutyrate; AcAc, Acetoacetate; KBs, Ketone bodies; TCA, Tricarboxylic acid cycle; CHO, Carbohydrate; HDAC, Histone deacetylases; BDNF, Brain-Derived Neurotrophic Factor; NLRP3, NOD-, LRR- and pyrin domain-containing protein 3 (inflammasome). (Some elements of this figure were created with BioRender.com; Paoli, A. (2026) https://BioRender.com/uc0m84r; https://BioRender.com/ehk3xdu).

## 7 Safety, limitations and research priorities

A preventive model of mild ketosis must be stated cautiously. Long-term trials demonstrating that recurrent mild ketosis prevents cognitive decline are not yet available. Strict ketogenic diets can be difficult to sustain and may cause gastrointestinal symptoms, constipation, micronutrient inadequacy or undesirable lipid responses in susceptible individuals. MCT commonly produce dose-dependent gastrointestinal discomfort, and ketone esters are limited by cost, taste and tolerability. In older adults, poorly designed dietary restriction may compromise energy or protein intake, which is especially relevant for preserving lean mass.

The preventive hypothesis therefore should not be translated into a universal recommendation for chronic ketogenic dieting. A more conservative and physiologically grounded approach is to study the effects of brief, repeated ketone elevations induced by feasible lifestyle strategies: overnight fasting, time-restricted eating, exercise performed with moderate carbohydrate availability, modest carbohydrate restriction, MCT-containing meals or carefully dosed exogenous ketones. These strategies may differ in efficacy and safety, and they should not be assumed interchangeable.

Future studies should move beyond the simple comparison of ketogenic versus non-ketogenic diets. Trials in healthy and middle-aged populations should quantify ketone exposure patterns: peak BHB, area under the curve, duration above baseline, frequency of ketone excursions and the metabolic conditions in which they occur. These measures should be related to cerebral substrate utilization, MCT expression or function where possible, cerebral blood flow, mitochondrial biomarkers, BDNF, inflammatory markers, sleep, mood, cognitive trajectories and metabolic phenotype. Baseline insulin sensitivity, habitual carbohydrate intake, cardiorespiratory fitness, age and body composition are likely to modify the response (5, 8, 9, 15, 48).

The key experimental question is no longer only whether ketones improve cognition acutely. It is whether maintaining the capacity to generate, transport and oxidize ketones helps preserve the brain’s metabolic repertoire across aging. This shifts the endpoint from short-term enhancement to long-term resilience.

## 8 Conclusions and future perspectives

Ketone bodies occupy an unusual position at the interface of nutrition, metabolism and neuronal signaling. Their traditional description as emergency fuels generated when carbohydrate availability is low is correct but incomplete. BHB and AcAc provide oxidizable carbon to the brain, while BHB participates in pathways relevant to gene regulation, inflammation, oxidative stress, neurotrophic activity and cellular resilience.

The strongest clinical rationale for ketone interventions remains in conditions characterized by cerebral glucose hypometabolism, such as MCI and early AD (. However, the more original implication is preventive. If ketones are normal metabolites that rise during fasting, exercise and reduced carbohydrate availability, the near absence of such episodes in modern metabolic life may represent a loss of physiological variability. Repeated mild ketone elevations could help maintain the pathways required for cerebral substrate switching and may contribute to brain wellness by supporting metabolic flexibility.

This perspective moves the field beyond the question of whether ketones are a better fuel than glucose. The brain does not need a single ideal substrate; it needs metabolic options. The relevant question may be: have we lost the physiological occasions on which ketones are supposed to rise? Testing this question will require longitudinal studies integrating dietary pattern, fasting duration, exercise, ketone dynamics, cerebral metabolism and cognitive aging. Until then, intermittent mild ketosis should be considered a promising hypothesis for brain health and prevention, not a proven prescription.
